# List of Diseases from the 20th of May to the 20th of June; Being the Result of the Public and Private Practice of a Physician at the West End of the Town

**Published:** 1799-07

**Authors:** 


*List of Diseases from the 20th of May to the 20th of June; being the Result of the Public and Private Practice of a Physician at the West End of the Town.*

| ACUTE DISEASES. | No. of Cases. | | No of Cases. |
|---|---|---|---|
| Measles - - - | 11 | Epilepsy - - | 2 |
| Hooping-cough - - | 3 | Chorea - - | 1 |
| Scarlatina - - - | 2 | Paralysis - - | 2 |
| Catarrh - - | 8 | Dyspepsia - - | 11 |
| Acute Rheumatism - - | 6 | Gastrodynia - - | 7 |
| Inflammatory Sore-throat - | 4 | Enterodynia - - | 5 |
| Pneumonic Inflammation - | 4 | Bilious vomitting & Diarrhœa | 12 |
| Peritoneal Inflammation - | 3 | Jaundice - - - | 3 |
| Volvulus - - | 1 | Chlorosis - - | 4 |
| Ophthalmia - | 3 | Menorrhagia - | 8 |
| Erysipelas - - | 2 | Abortion - - | 3 |
| Synochus, or Summer-fever | 6 | Amenorrhea - | 4 |
| Contagious malignant Fever | 6 | Fluor Albus - - | 3 |
| Apoplexy - | 2 | Emansio - - | 2 |
| Epistaxis - - | 3 | Scirrhus Uteri - - | 1 |
| Hæmoptoe - - | 4 | Scirrhus of the Liver - | 2 |
| Hæmatemesis - - | 2 | Dropsy - - | 3 |
| Intestinal Hæmorrhagy - | 2 | Scrofula - - | 6 |
| Renal Hæmorrhagy - - | 2 | Tabes Mesenterica - | 4 |
| Tertian - - - | 1 | Hydrocephalus - | 1 |
| Hectica - - | 3 | Worms - - | 3 |
| Child-bed and Milk-fever | 3 | Hæmorrhoids - | 2 |
| Acute Diseases of Infants | 12 | Prolapsus Ani - - | 1 |
| CHRONIC DISEASES. | | Scaly Tetter - - | 4 |
| Asthenia - - | 43 | Nettle-rash - - | 1 |
| Cough and Dyspnœa - | 31 | Pompholyx - - | 1 |
| Chronic Rheumatism - | 20 | Impetigo - - | 3 |
| Lumbago and Sciatica - | 7 | Ecthyma - - | 2 |
| Pleurodyne - | 4 | Itch and Gratelle - | 12 |
| Pulmonary Consumption | 12 | Gutta Rosacea - - | 4 |
| Cephalœa - | 6 | Porrigo - - | 3 |

The series of incongruous complaints stated in the above list, is an usual effect, in our climate, of hot sun-shine, struggling with the piercing east and north-east winds, which mostly prevail from the commencement of spring, till the summer solstice. Indeed, the diseases of winter, spring, and summer, seem to have been crouded together within the last six weeks; and must have fully enabled the faculty to meet the payment of the income-tax.

Among *contagious epidemic* disorders, the measles have lately taken the lead; but from an inspection of the list it will appear, that *inflammatory* and *hæmorrhagic* complaints formed the most extensive set of acute diseases; the latter series being particularly violent and obstinate, and in many instances fatal. Thus far we can sympathize in this peaceful island, with the unhappy sufferers sufferers on the plains of Italy and Helvetia: but as the above stated diseases of our fellow-citizens will probably be terminated by the *asthenic languor* of July; so, we will hope, the *almast paralytic asthenia* of our enemies may prevent the further effusions of blood on the continent, and be the means of restoring health, peace, and happiness, to all the nations of Europe.

